# Innovative Hydroxyapatite-Coated Titania Nanotubes for Dental Implant Surface Enhancement

**DOI:** 10.4317/jced.62626

**Published:** 2025-06-01

**Authors:** Parkavi Arumugam, Bianca Princeton, Pradeep Kumar Yadalam, Carlos M. Ardila

**Affiliations:** 1 Department of Periodontics, Saveetha Dental College and Hospital, Saveetha Institute of Medical and Technical Sciences, Saveetha University, Chennai, Tamil Nadu, India; 2 Basic Sciences Department. Biomedical Stomatology Research Group, Faculty of Dentistry Universidad de Antioquia, UdeA, Medellín, Colombia

## Abstract

**Background:**

The present study aimed to develop novel hydroxyapatite-coated titanium dioxide or titania nanotubes (TNTs) as a surface modification on titanium dental implants and analyze their surface, chemical properties, biocompatibility, and corrosion resistance.

**Material and Methods:**

The titanium implant surface was treated with 1 ml of Kroll’s reagent, 5 ml of nitric acid, 1.5 ml of sulfuric acid, and water for 10 seconds to allow etching of the surface. The etched surface was then anodized to create a layer of titanium dioxide, which, on treatment with 1wt% of hydrofluoric acid in water under the anodization process with 100 volts for 1 hour at room temperature, led to the formation of TNTs. The nanotube surface was then dipped in Hank’s solution, allowing hydroxyapatite deposition on the surface. After 7 days, the hydroxyapatite-coated TNTs (GROUP A) as a surface coating on titanium implants was characterized and compared with bare titanium implants (Group B).

**Results:**

The material characterization showed successful development of hydroxyapatite-coated TNT formation on titanium implant surface, which supported cell adhesion, proliferation, and migration, similar to uncoated titanium surfaces. No statistically significant difference in the percentage of cell viability was noted between Groups A and B at any time point, with the highest percentage of cell viability with a mean of 93.20 +/- 4.324 for Group A and 94.00 +/- 6.205 for Group B noted at 72 hours, with a p-value of 0.21. Corrosion testing showed the coating’s higher corrosion potential and reduced corrosion density compared to uncoated titanium surfaces with the bode phase angle approaching 1, suggesting its potential for better clinical outcomes.

**Conclusions:**

The hydroxyapatite-coated TNTs have good surface, chemical corrosion-resistant properties, and optimal biocompatibility. Further in vivo studies are warranted to assess the osteogenic and antimicrobial properties, as well as the clinical efficacy, of this coating.

** Key words:**Dental implant, hydroxyapatite, titania nanotubes, biocompatibility, corrosion, osseointegration.

## Introduction

Advancements in healthcare have enhanced life expectancy worldwide. As many aging individuals face edentulism, optimal tooth replacement becomes crucial. Among available options, dental implants are preferred over other prostheses for their superior chewing efficiency and aesthetics. Various shapes and sizes of titanium dental implants are utilized because of their exceptional osseointegration and passive characteristics. Nevertheless, these implants can also lead to biological issues, such as peri-implant infections and inadequate support for osseointegration (1-3). To overcome these issues, surface coatings and modifications are being explored and developed (4,5) .

Nano-biomaterials employ nanostructured materials such as polymers, ceramics, metals, and self-assembled materials using top-down or bottom-up approaches for generation, characterization, and modeling for biomedical applications. Nanostructures are materials within the nanometer scale and have one or more dimensions. They can be categorized by dimensionality as one, two, or three-dimensional; by morphology as nanoparticles, nanotubes, nanospheres, nanowires, nanosheets, and nanoribbons; by composition as either organic or inorganic; and by distribution as dispersed or aggregated. Among these, metal oxide nanostructures are particularly favored for their promising antimicrobial, osteogenic, and semiconducting properties (6,7).

Recently, the development of nanostructures on titanium surfaces has gained much attention due to their increased surface-to-volume ratio, thereby greatly improving osseointegration. Additionally, because the nanostructures are created directly from the implant’s base metal, they sidestep the delamination problems associated with bioactive surfaces. Various types of titanium nanostructures, including nanotubes, nanowires, nanosheets, and nanoribbons, are under investigation. Research has demonstrated enhanced mechanical properties and biocompatibility with titanium nanostructures (8).

Titania, also known as titanium dioxide nanotubes (TNTs), are elongated structures made from titanium dioxide that exhibit versatile ceramic characteristics, including chemical inertness and high stability. The electrochemical anodization method has become the standard for synthesizing these nanotubes due to its simplicity, affordability, scalability, and lower toxicity. This technique allows for enhanced control over the synthesized nanotubes’ shape, structure, and morphology by adjusting anodization parameters such as voltage, pH, and concentration. Additionally, TNTs demonstrate promising antimicrobial and osteogenic properties. Research indicates that TNTs on titanium surfaces enhance osseointegration, cell adhesion, proliferation, and antimicrobial activity properties (9). Adding calcium phosphate-based ceramics would be beneficial in further improving the osteo-promotive effect of the TNT surface.

Hydroxyapatite is a widely used bone graft material that has osteoconductive characteristics. This compound consists of calcium and phosphorus in a 1.67 ratio and is a key structural element of bone tissue. We hypothesize that hydroxyapatite-coated TNTs as a surface modification of titanium dental implants would enhance the surface, biocompatibility and corrosion resistance properties of the implant for improved cell adhesion, proliferation, and osseointegration. To our knowledge, no previous research has examined the potential of hydroxyapatite-coated TNTs for implant surface modification to boost osseointegration. The objective of the present study was to develop novel hydroxyapatite-coated TNTs as a surface modification of titanium dental implants and analyze their surface (Scanning Electron Microscope analysis), chemical properties (Energy dispersive X-ray and Fourier Transform Infrared Spectroscopy analysis), biocompatibility (Confocal analysis), and corrosion resistance (Corrosion analysis).

## Material and Methods

This study utilized analytical-grade chemicals sourced from Sigma-Aldrich and Merck Group, Germany, ensuring experimental precision and reliability. The chosen commercially pure titanium grade 2 plates, measuring 20×15×2 mm, were ideal for biological applications, especially for implants, and were sourced from Ti Anode Fabricators Pvt Ltd in Chennai, India. Biocompatibility tests were conducted using MG63 cells from the National Centre for Cell Science in Pune, India. Hi Media Laboratories Private Limited in Thane, India, provided growth media, staining solutions, and cell culture supplements. The titanium implant surface underwent treatment with 1 ml of Kroll’s reagent, followed by 5 ml of nitric acid, 1.5 ml of sulfuric acid, and water for 10 seconds to facilitate surface etching. Following the etching, the titanium implant surface was subjected to oxidation through anodization to create a titanium oxide surface layer. For this, a 1 wt% hydrofluoric acid solution in water was employed as the electrolyte, establishing an acidic environment that promotes the dissolution of the titanium oxide layer during anodization, ultimately leading to nanotube formation. Fluoride ions in the electrolyte are crucial for generating self-organized nanotube structures. Anodization was performed by applying a voltage of 100 volts for one hour at room temperature. Afterward, the TNTs-coated implant surface was immersed in Hank’s solution to encourage hydroxyapatite deposition. After seven days, the hydroxyapatite-coated TNT surface on titanium implants was characterized.

Material characterization: The study investigated the characteristics of hydroxyapatite-coated TNTs to enhance the biocompatibility and osteointegration of titanium implants. To evaluate surface features and chemical composition, advanced analytical methods were employed, including Field Emission Scanning Electron Microscopy (FE-SEM, JSM IT800 (JEOL, Tokyo, Japan), Energy Dispersive X-ray (EDX), and Fourier Transform Infrared Spectroscopy (FTIR) (Bruker alpha two). These results are vital for assessing the material’s appropriateness for medical applications.

Biocompatibility analysis: The study examined the biocompatibility, cell adhesion, and growth patterns of hydroxyapatite-coated TNTs as a surface modification for titanium implants, utilizing the MG63 cell line. MG63 cells were selected due to their relevance for osteogenesis studies *in vitro*, providing important insights into cell-material interactions. The experimental group comprised hydroxyapatite-coated TNTs on titanium implants (Group A), while the control group featured uncoated titanium implants (Group B). The culture medium was prepared with Dulbecco’s Modified Eagle’s Medium (DMEM), supplemented with 10% fetal bovine serum (FBS) and 1% penicillin. Cells were cultured until reaching approximately 80% confluency, then were detached from flasks and plated onto the implant samples at a density of 10,000 cells per cm². To evaluate cell attachment, spreading, and proliferation, the cells underwent a staining process using rhodamine B, acridine orange, and combinations of staining techniques. Following staining, the cells were washed with phosphate-buffered saline (PBS) and fixed with 4% paraformaldehyde for imaging. The stained and fixed samples were placed on glass slides and analyzed through the Confocal Laser Scanning Microscope LEICA DMI8. A longitudinal assessment at 24, 48, 72, 96, and 120 hours enabled a thorough evaluation of the biocompatibility of the implant coating compared to the uncoated titanium controls. The study used a small sample size of 5 per group to detect the differences, aiming for statistical significance. The sample size was chosen due to its high reproducibility and low variability in *in vitro* settings compared to *in vivo* models. The 5% alpha error and 80% beta error were maintained, along with the controlled *in vitro* conditions and alignment with prior research standards, that justified the small sample size.

Corrosion analysis: Corrosion resistance was assessed by immersing the implants in simulated body fluid (SBF) for 45 minutes, maintaining an open circuit potential throughout the immersion process. Electrochemical impedance spectroscopy (EIS) measurements were conducted to evaluate the material’s protective coating and its ability to resist corrosion. After stabilization, an amplitude of ±10 mV was employed for the impedance analysis. The impedance spectra were recorded over a frequency range of 0.01 Hz to 200 kHz. Additionally, a polarization study was performed to assess the electrochemical behavior of the titanium implants and their corrosion resistance in simulated biological environments at a scan rate of 1 mV/sec between -1V and 0.05V.

Statistical analysis: Statistical analysis was performed using IBM SPSS Statistics for Windows, with an unpaired t-test applied to compare the biocompatibility of the hydroxyapatite-coated TNT surface against the bare titanium surface. The significance levels were considered statistically significant if the *p-value* was less than 0.05 (*p*<0.05), ensuring that the differences observed between the two surfaces in terms of cell viability, growth, and activity were not due to random chance but rather reflective of the true effects of the coating.

## Results

- Material characterization

**Figure 1:**
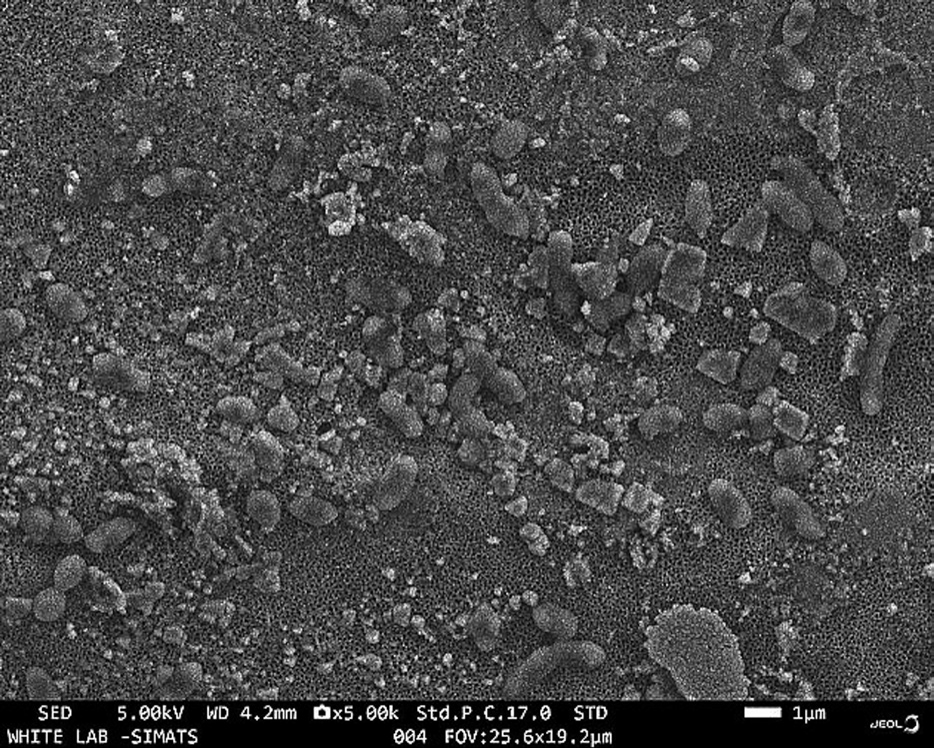
SEM analysis of hydroxyapatite-coated TNTs on titanium implants showing a layer of the hydroxyapatite crystals on a layer of hollow tubes arising from the titanium implant.

SEM analysis: On SEM (Scanning Electron Microscopy) analysis (Fig. 1), the developed hydroxyapatite-coated TNTs surface revealed a rough surface with tubular openings as a base over which hydroxyapatite crystals were attached. This proved the successful development of TNTs from the base metal surface and the attachment of the hydroxyapatite crystals to the developed nanotubular structures. The nanotubes would greatly improve the surface area for cell interaction and bone deposition. Moreover, the hydroxyapatite crystals would provide osteogenic cues for the proliferating cells. The results showed the development of a surface conducive to cell adhesion, differentiation, and proliferation.

EDX and FTIR analysis: The results from EDX (Energy Dispersive X-ray) analysis provide significant insights into the elemental composition of the material being examined. Titanium and oxygen emerged as the predominant constituents, with weight percentages of 65.1% and 30.7%, respectively. These high percentages suggest that titanium is the primary base metal of the implant, crucial for its structural and mechanical integrity, while oxygen likely originates from the titanium nanotubes (TNTs) structured at the surface of the implant. This implies a strong correlation between the elemental composition and the intended functionality of the implant, highlighting the importance of titanium in biomedical applications due to its biocompatibility and resistance to corrosion. Moreover, the EDX analysis revealed trace amounts of calcium and phosphorus at 3.9% and 0.3% weight percentages. The presence of these elements is particularly noteworthy as they may signify the successful deposition of the hydroxyapatite layer atop the TNTs. Hydroxyapatite is a bioceramic material that closely resembles the mineral component of bone, thereby enhancing the implant’s ability to promote osseointegration, essential for the long-term stability of implants within biological systems. Complementing the EDX findings, Fourier Transform Infrared (FTIR) analysis supported these conclusions. The FTIR spectrum displayed multiple characteristic absorbance peaks that correlate with the presence of various functional groups. The broad absorbance band observed between 3600-3000 cm-1, along with a distinct peak at 1643 cm-1, reinforces the existence of hydroxyl functional groups. These hydroxyl groups are likely instrumental in promoting the material’s bioactivity. Furthermore, absorbance peaks at 1439 cm-1 and 1018 cm-1 indicated the presence of carbonate and phosphate functional groups, respectively. These findings bolster the argument for the calcium phosphate presence as hydroxyapatite crystals within the material, which is vital for supporting cellular functions associated with bone mineralization. Lastly, the FTIR analysis also identified absorbance peaks at 769 cm-1 and 642 cm-1 that corroborated the interactions of Ti-O-Ti and Ti-O bonds. This further validated the presence of titanium oxide in the composite, affirming the role of titanium as the base metal. The detection of these peaks is significant as it not only indicates the various chemical states of titanium present but also highlights the material’s potential interactions with biological environments upon implantation ( Table 1).

**Figure 2:**
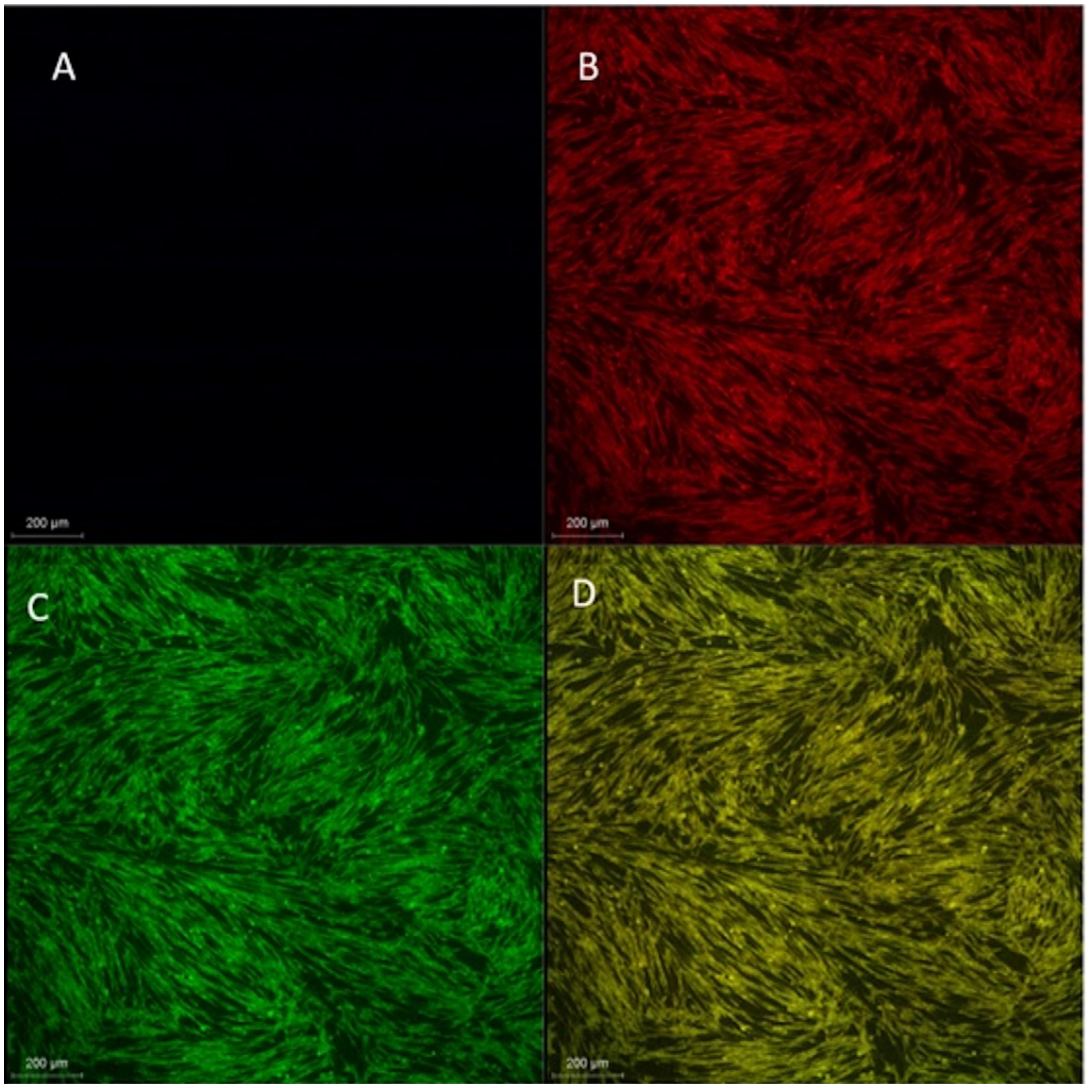
Biocompatibility analysis of hydroxyapatite-coated TNTs on titanium implants using confocal microscope. A- blank; B- Rhodamine B staining; C- Acridine Orange staining; D- combination staining.

**Figure 3:**
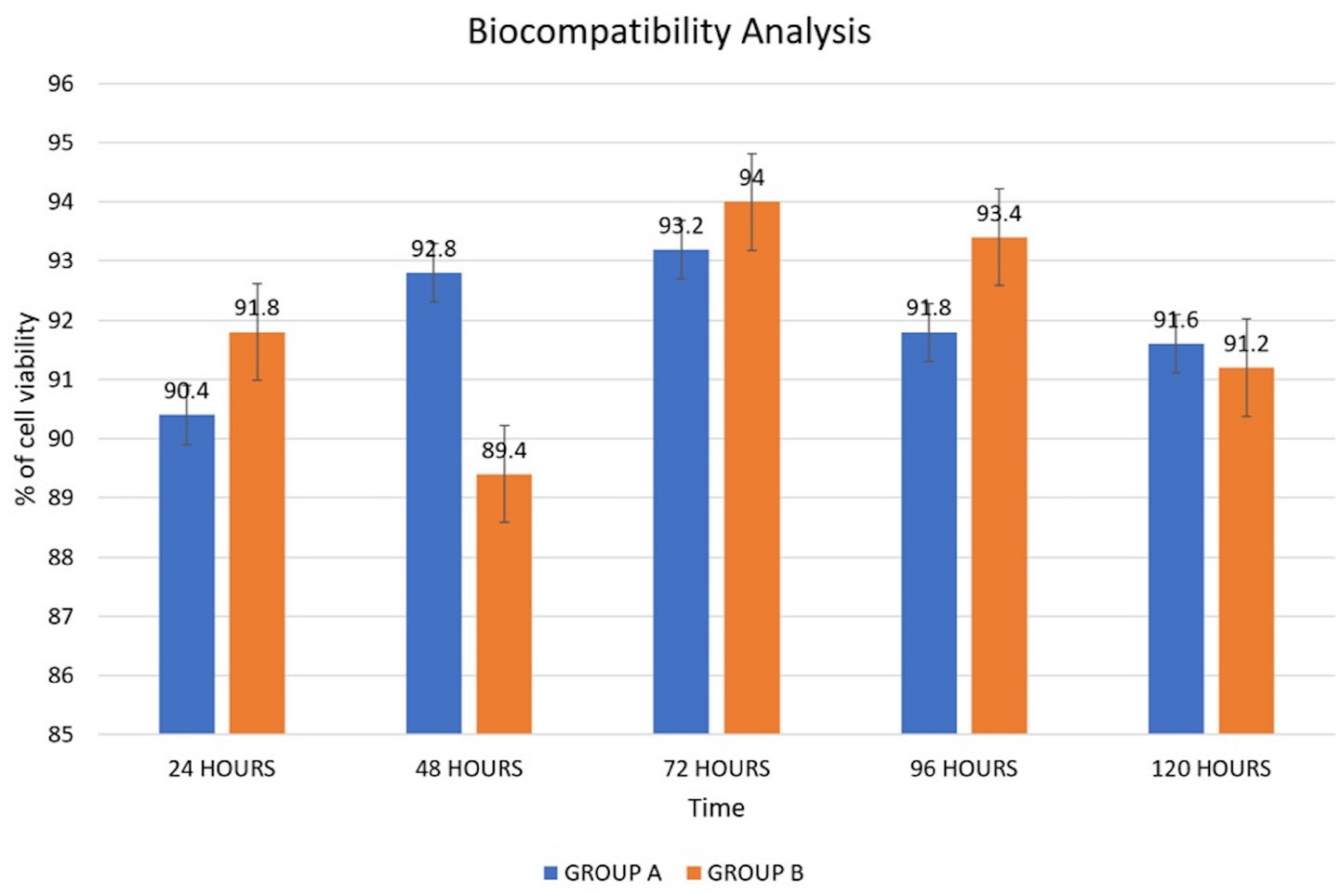
Biocompatibility analysis of hydroxyapatite-coated TNTs on titanium implants - Statistical analysis.

Biocompatibility analysis: The implant surface modification (GROUP A) was tested *in vitro* to evaluate its biocompatibility. A bare titanium implant surface (GROUP B) served as a control. MG63 osteoblastic cells were cultured on the surface, and their viability, adhesion, and growth characteristics were assessed. As shown in Figure 2, the results indicated that the modified surface promoted effective cell adhesion and supported the formation of multiple layers of cells, which is essential for bone-implant integration. The cells displayed well-organized cytoskeletal structures, indicative of metabolic activity, as confirmed by Rhodamine B staining. The cells also exhibited extended filopodia, thin projections related to cell migration and intercellular communication. These extended filopodia reinforce the idea that the implant surface modification did not impede cell movement or the formation of intercellular connections, which are vital for tissue integration in orthopedic and dental implants. The cells were further stained with Acridine Orange, confirming their health and survival on the implant surface. The results indicated no statistically significant difference between the two groups (Fig. 3,  Table 2), suggesting that the implant surface modification did not adversely affect cell survival or function compared to the traditional titanium surface at any time point. These findings imply that the developed implant surface modification (GROUP A) demonstrates good biocompatibility, supporting cell adhesion, proliferation, and migration similarly to the uncoated titanium surface. The surface modification’s ability to enhance cell attachment and growth highlights its potential for improving the integration of titanium implants with surrounding bone tissue, making it a promising candidate for future implant applications.

**Figure 4:**
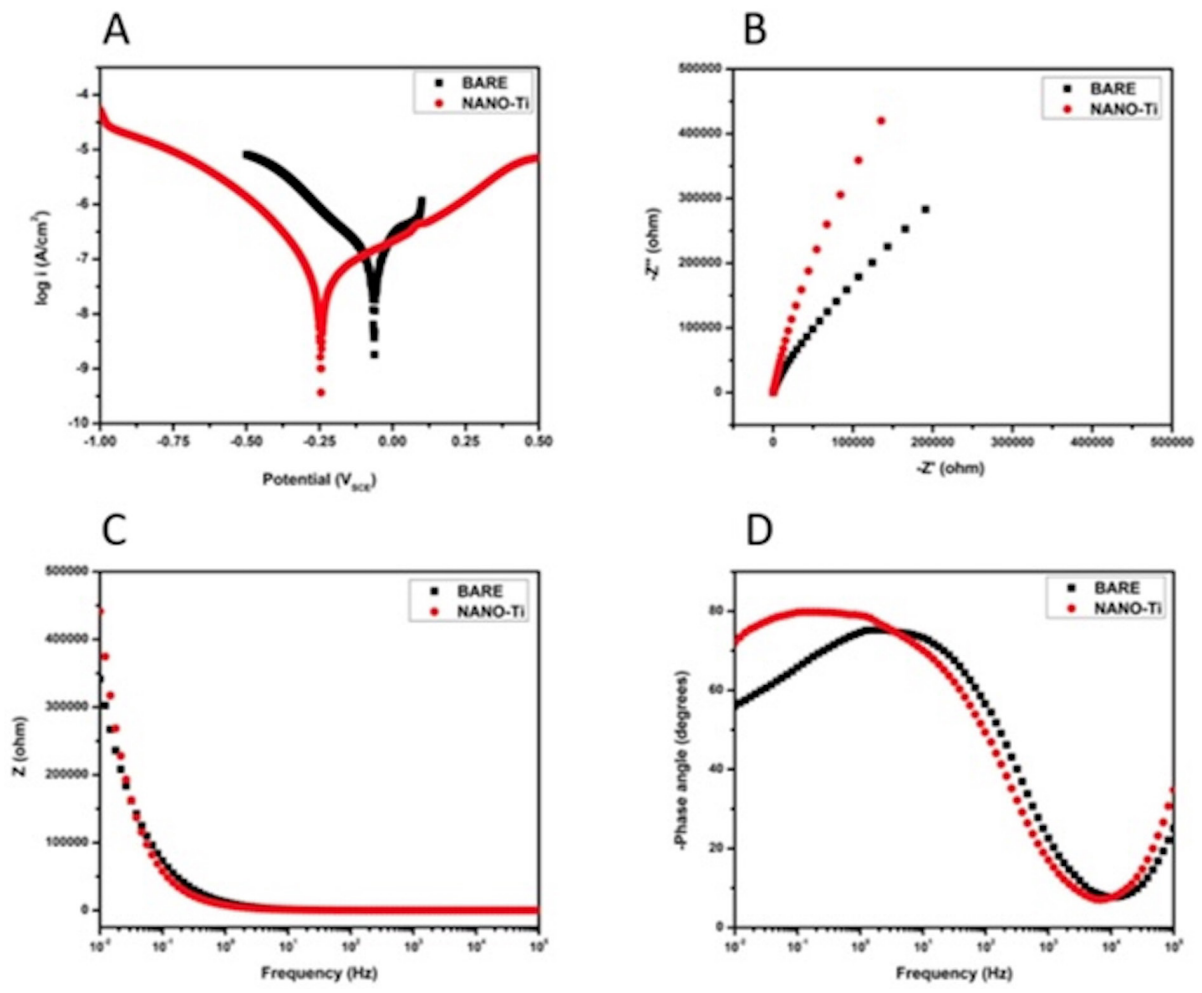
Corrosion analysis of the developed implant coating, A- potentiodynamic polarization; B- Nyquist plot; C- bode impedance; D- bode phase angle.

Corrosion analysis: Corrosion testing was meticulously conducted using simulated body fluid to evaluate and compare the electrochemical behavior of the modified surface in comparison to that of the bare titanium surface. The detailed findings of this investigation are illustrated in Figure 4. Results indicated that GROUP A, which underwent surface modification, demonstrated a notably higher corrosion potential (Ecorr), which shifted positively when compared to GROUP B. This observation suggests that the developed surface modification possesses enhanced thermodynamic stability relative to the bare titanium, indicating a promising advance in the field of material science. Moreover, GROUP A exhibited a significant reduction in current density (Icorr) at lower current levels when juxtaposed with the results from GROUP B, thereby reinforcing the assertion of superior corrosion resistance attributed to the surface modification. The comprehensive analysis of the Nyquist plot further characterized the electrochemical properties, revealing a substantial charge transfer resistance that implies GROUP A’s reduced susceptibility to corrosive environments compared to GROUP B. At lower frequencies, the bode impedance modulus for GROUP A prominently exceeded that of GROUP B, reflecting its superior performance in terms of impedance characteristics. Additionally, the bode phase angle for GROUP A approached unity, which is indicative of the modified surface behaving similarly to a constant phase element. These findings collectively support that the developed surface modification not only demonstrates robust thermodynamic stability but also exhibits superior impedance characteristics, significantly enhancing its resistance to corrosion phenomena. The corrosion analysis results provide compelling evidence of the surface modification’s effectiveness, positioning it as a viable alternative for applications requiring improved material durability in corrosive environments.

## Discussion

The effort to improve the osseointegration has been made through implant surface modifications. Various physical, chemical, and biological methods, such as acid etching, sand blasting, plasma spraying, surface coatings, anodization, and bioactive coatings, have been employed to achieve the same (10,11). The present study developed a novel surface modification of hydroxyapatite-coated titanium nanotubes on titanium implants to enhance osseointegration outcomes. The SEM analysis revealed a surface with well-defined nanotube development and tubular openings. The nanotubes also displayed hydroxyapatite crystals adhered to the surface of the implant. The tubular TNT structures mimic the trabecular architecture of bone. Furthermore, the presence of hydroxyapatite provides compositional similarity to bone structures, offering osteogenic sites for calcification and bone deposition. Consequently, this surface facilitates the adhesion of mesenchymal stem cells, their differentiation, and proliferation into osteogenic lineage cells. Studies have indicated that nano-surfaced materials like TNTs replicate bone’s natural hydroxyapatite and collagen components, enhancing osteogenesis (12). Their nano-topography allows for optimal interactions for osteogenesis, attracting proteins like vitronectin and fibronectin. This promotes osteoblast adhesion and proliferation.

EDX analysis revealed a successful coating of hydroxyapatite crystals over the TNT surface. The FTIR analysis revealed the presence of absorbance peaks that corresponded to Ti-O and Ti-O-Ti bonding. Other bands that corroborated the presence of O-H stretching, O-H bending, P-O interactions, and R-C=O interactions could also be observed. These results show the presence of titanium dioxide and calcium phosphate in the form of hydroxyapatite crystals. The results of this study are similar to those that assessed TNTs (13).

The implant surface modification was evaluated for biocompatibility by culturing MG63 osteoblast-like cells on the modified surface. The results showed a homogeneous distribution of spindle-shaped, viable osteoblast-like cells across the surface, supporting cell attachment and forming a well-organized layer of cells. The cells exhibited a well-developed cytoskeleton, including extended filopodia, which indicates active movement and potential interaction with other cells. The implant surface modification did not adversely affect cell health and supported cellular functions such as attachment, proliferation, and differentiation, proving that the developed implant surface was biocompatible. Similar results were observed in a study by Gongadze *et al*., who suggested that negatively charged titanium surfaces attract osteoblasts through charged proteins with quadrupolar internal charges (14). They also stated that the attraction between fibronectin molecules and titanium surfaces, facilitated by cations, is more effective when the surface charge density is high, which promotes osteoblast adhesion through integrin interaction.

Furthermore, corrosion analysis on the modified surface confirmed the material’s suitability for implant applications. It was thermodynamically sTable, maintaining structural integrity and not degrading under simulated biological conditions. The modified surface also demonstrated improved corrosion resistance, supporting its potential for use in biological environments. These results are in accordance with other studies that assessed the behavior of hydroxyapatite and titanium nanotube-coated implant surfaces (15). Another study on resveratrol-coated TNT stated that the coating significantly promoted osteogenic indicators in BMSCs, including ALP production, calcium deposition, and osteogenesis-related gene expression like alkaline phosphatase (Alp), Osteocalcin (OCN), and Osteopontin (OPN) (16). They further stated that the coating down-regulated Nuclear Factor Kappa Beta (NF-κB) phosphorylation, suggesting it could inhibit inflammation and promote osteogenesis, making it an effective implant surface for improving osseointegration ability.

TNTs have gained preferential attention due to their significant properties like greater surface area, catalytic activity, better chemical stability, and behavior in biological fluid systems. Their tubular hollow structure provides a biomimicry effect for cell attachment and differentiation. They are also ideal for the loading and releasing of drugs and growth factors of interest. The effect of TNT’s on the osteoblast cell interaction can be explained based on the concept proposed by Wu *et al*. who categorized osseointegration into four stages namely protein adsorption, inflammatory response, cell adhesion, and the angiogenic-osteogenesis stage (8). They stated that protein adsorption during implant placement was found to influence tissue response, with surface charge and wettability being observed to affect adsorption ability. The quality of adsorbed proteins was considered more crucial than the quantity of the adsorbed proteins. Adsorbed proteins such as fibronectin and vitronectin were found to act as extracellular signals for cell cytoskeleton organization, influencing the adhesion and spreading of osteoblast-like cells and other cells. TNTs surface on titanium implant creates a hydrophilic implant surface with a greater surface area which is conducive for protein adsorption noted in the crucial early-stage post-implant placement. Studies have proven that TNTs-coated titanium implant surface were more hydrophilic and adsorbed more proteins than bare titanium surface (17).

In the second stage that is dominated by an inflammatory phase, leading to platelet aggregation and clot formation on the implant surface, evidence proves that TNTs-coated surface showed accelerated platelet adhesion and spreading, causing a greater release of growth factors (8,18). Research has also shown that the TNTs have an ability to modulate the macrophage polarisation to M1 or M2 phenotype (18). A study found that macrophages cultured on 90 nm diameter TNTs showed up-regulation of the peroxisome proliferator-activated receptor (PPAR) and Ras homolog gene family, member A (RhoA)/Rho-associated protein kinase (ROCK) signaling pathways, while down-regulation of M1-polarization pathways like Mitogen activated kinases (MAK), Adenosine Monophosphate-activated protein kinase (AMPK), Tumour necrosis factor (TNF), and nucleotide-binding oligomerization domain (NOD)-like receptors, which would reduce the pro-inflammatory phase and direct the tissue response towards an anti-inflammatory and pro-healing phenotype (8,19). Furthermore, the conFiguration of TNTs have been shown to enhance focal adhesion complex and F-actin stability, while lateral spacing influences osteoblast behaviors, with 80 nm nanotubes showing less spreading (8).These structures were found to intensify cell adhesion and to facilitate contact osteogenesis on implant surfaces.

Research has shown that pore diameter, length, and direction of alignment of the TNTs have considerably affected cell adhesion and differentiation (9). A study that developed TNTs with varying pore diameters ranging from 30nm to 100nm stated that on human mesenchymal stem cell (hMSC) augmentation, there was an increase in osteoblastic differentiation that was induced by only the geometric cues, in the absence of any osteogenic medium (20). It was observed that TNTs with smaller pore diameters allowed for enhanced cell adhesion, while TNTs with larger pore diameters caused enhanced osteoblast differentiation. The larger diameter TNTs cause the elongation of stem cells, causing cytoskeletal stress and leading to increased cell differentiation. The effect of pore diameter found that nanotubes with small diameters stimulated the highest adhesion.

TNTs have been found to promote endothelial cell spreading and migration in vascular stents, potentially enhancing angiogenesis (21). They also directly affect osteoblasts, regulating cell behaviors and enabling osteoblasts to stretch well with filopodia growing into nanotube pores. The surface nano-pattern plays a guiding role in cell adhesion and spreading, potentially enhancing angiogenesis and osteogenesis. Researchers used TNTs’ adjustability to explore the most suiTable diameter for cell adhesion and osteogenic differentiation. Osteoblasts cultured on TNTs with 80 nm spacing exhibited a significant increase in alkaline phosphatase (ALP), osteopontin (OPN), and osteocalcin (OCN) expression, indicating high osteogenic activity (22).

The length of TNTs significantly impacts the osseointegration process, with longer TNTs providing more surface area and a larger topographical cue for cells, enhancing osteoblast adhesion, proliferation, and differentiation (9). They influence cell morphology and alignment, promoting more organized tissue formation and enhancing osseointegration. They also increase the number of adsorbed proteins like fibronectin and collagen, which are crucial for cell attachment and signaling. However, long TNTs may introduce mechanical challenges during early healing (9). The bioactivity of TNT surfaces can be affected by their length, with longer TNTs potentially enhancing ion release and supporting bone regeneration. A study reported that TNTs of 100 µm length could load ~2.5 mg/cm2 of the drug, which showed sustained release for 3 weeks (23). Ultimately, the optimal length of TNTs should be tailored to the specific application, considering material properties and the biological environment.

Research has shown that TNTs, compared to bare titanium surfaces, showed an increased mineralization rate. An *in vitro* study compared human osteoblast cells on TNTs and tantalum-coated TNT surfaces (24). The mineralization rate was three times faster on TNTs and twice as fast on micro-textured tantalum. The tantalum surface promoted a 30% faster mineralization rate and bone-nodule formation than bare TNTs. This evidence proves that adding osteogenic cues in the form of bone grafts and growth factors onto the TNT surface further augments the coatings’ cell differentiation and mineralization potential. In the present study, adding hydroxyapatite crystals over the TNT surface may perpetuate the implant’s osseointegration ability. In another study, a novel coating of TNTs loaded with polydiamine and silver cross-linked basic fibroblast growth factor (PDA/Ag/bFGF) was developed on the titanium dental implant surface (25). They stated that the developed implant surface had good surface properties and slow bioactive bFGF release. Cross-linking silver with bFGF allowed for anti-inflammatory and anti-bacterial properties, enhancing osteogenic differentiation. Cowden *et al*. studied the adipose-derived mesenchymal stem cell (ADSC) behavior response on TNT surfaces. They stated that ADSC proliferation and differentiation were affected by the size of TNTs (26). Zhao *et al*. developed extracellular vesicles (EV) derived as exosomes from mesenchymal stem cells and coated them over TNTs (27). It was observed that EV hybrid TNTs showed significantly reduced inflammatory gene and protein expression with enhanced cell migration and osteogenic differentiation.

Recent evidence has shown that TNTs also exhibit potential antimicrobial activity. TNTs on titanium surfaces reduced Staphylococcus epidermidis colonization compared to polished or acid-etched titanium surfaces (28). The larger nanotubes had the highest antimicrobial effect, possibly due to their hydrophilic surface’s negative charge attaching to osteoblasts and repelling microbes, reducing biofilm build-up and infection resistance. A study that analyzed TNTs on porous titanium scaffolds that were further treated with silver nitrate showed effective prevention of biofilm formation and decreased planktonic bacteria, with the antimicrobial effects continuing for two weeks (29). TNTs, due to their high surface hydrophilicity and unique nano-topographical structure, effectively inhibit bacterial adhesion on implants, reducing biofilm formation through “charge repulsion” of negatively charged cell membranes. This also further proves the antimicrobial potential and the increased scope of doping the TNTs with antimicrobial agents, growth factors to achieve good clinical outcomes.

Furthermore, a recent systematic review that assessed the osseointegration potential of hydroxyapatite implant coatings, concluded that hydroxyapatite surface treatment enhances titanium dental implant osseointegration by promoting protein absorption, adhesion, and bone cell proliferation through proper adhesion methods (30). The results of our study are in accordance with the scientific consensus. Also, it has been concluded that bioactive surface changes enhance osseointegration and implant lifespan, however, optimising the doses and combinations are required for optimal results (10). However, more *in vitro* and pre-clinical studies are needed to assess their potential for preventing peri-implant infections. Hence, based on the scientific literature and comparison of our results with the evidence available, it can be concluded that though various implant surface modifications and biomaterial coatings have been developed, further long-term research is required to claim superiority of these innovations.

In the present study, though the developed implant surface coating has shown promise, it is not without its limitations. The developed TNTs were not optimised for pore diameter, tube length, tube orientation and intertubular space. Moreover, achieving a uniformly thick optimised hydroxyapatite coating on the TNTs surface that does not impair the nano-topographical features, mechanical strength and implant performance is needed. Also, the propensity for hydroxyapatite to undergo faster degradation, requires tailoring the degradation rate to one that is in accordance with the rate of osseointegration. This issue can be worked out in the future, by developing polymeric coatings on hydroxyapatite to tailor the degradation rate and by improving the functionalization of the hydroxyapatite layer by doping it with biomolecules and growth factors. It is safe to say that the results of this study are too preliminary, to claim superiority over the available industry standards. Further *in vitro* and *in vivo* studies should be conducted to analyse the impact of pore diameter and length on the mineralization and the antimicrobial properties of the implant surface modification.

In conclusion, hydroxyapatite-coated TNTs on titanium implants have shown positive surface chemistry, chemical structure, optimal biocompatibility, and good corrosion resistance properties. The developed coating, may translate clinically as a biocompatible surface coating that will minimize adverse tissue reactions and reduce the risk of peri-implant inflammation and infections. The corrosion-resistant properties of the developed coating will reduce the risk of implant degradation, improving the long-term clinical viability of the implant. The nanotubular structure of the titanium surface will enhance vascularization around the implant site, facilitating better angiogenesis and osseointegration. Moreover, we propose that TNTs may aid in the osteogenic and antimicrobial properties of the implant surface owing to their physical structure, converting mechanical cues into intracellular signals. Adding hydroxyapatite over the TNTs would further add a synergistic effect on the mineralization effect and osseointegration process. Future research should focus on optimising the hydroxyapatite coated TNTs layer and analyse its mineralisation potential and antimicrobial effects.

## Conclusions

The hydroxyapatite-coated TNT surface modification on titanium implants has exhibited good surface, chemical, biocompatibility, and corrosion-resistant properties, with the potential for enhanced osseointegration outcomes. Future research should focus on long-term *in vivo* studies to determine the mechanical stability, osteointegration and antimicrobial potential over extended periods. Genetic and cellular mechanisms should be explored, and the nanotube structure should be precisely adjusted to optimize the osteointegration process. Clinical translation and standardization are necessary for hydroxyapatite-coated TNTs implants to gain widespread clinical acceptance. Combining hydroxyapatite-coated TNTs with other biomaterials could offer multi-functional implants that promote bone growth, prevent infection, or release therapeutic agents for accelerated healing.

## Figures and Tables

**Table 1:** EDX analysis of the developed implant surface coating.

Wave number (cm^-1^)	Bond	Functional group
Broad band around 3600-3000	O-H stretching	Hydroxyl group
1643	O–H bending vibrations	Hydroxyl group
1439	R-C=O	Carbonate group
1018	P-O interaction	Phosphate group
769	Ti-O-Ti interaction	Titanium oxide
642	Ti-O interaction	Titanium oxide

**Table 2:** Biocompatibility analysis of the hydroxyapatite-coated TNTs on titanium implants in comparison with bare titanium implants – student’s t test.

TIME	SAMPLE	N	MEAN (% OF CELL VIABILITY)	STANDARD DEVIATION	MEAN DIFFERENCE	P VALUE	95% CONFIDENCE INTERVAL	
							LOWER LIMIT	UPPER LIMIT
24 HOURS	GROUP A	5	90.40	5.459	-1.40	0.71	83.62	97.18
	GROUP B	5	91.80	4.494			86.22	97.38
48 HOURS	GROUP A	5	92.80	1.304	3.40	0.68	91.18	94.42
	GROUP B	5	89.40	1.817			87.14	91.66
72 HOURS	GROUP A	5	93.20	4.324	-0.80	0.21	87.83	98.57
	GROUP B	5	94.00	5.439			88.56	99.43
96 HOURS	GROUP A	5	91.80	6.140	-1.60	0.10	84.18	99.42
	GROUP B	5	93.40	3.362			89.23	97.57
120 HOURS	GROUP A	5	91.60	5.273	0.40	0.42	85.05	98.15
	GROUP B	5	91.20	4.438			85.69	96.71

## Data Availability

The datasets used and/or analyzed during the current study are available from the corresponding author.
